# Rectus femoris cross sectional area and timed up and go test potential useful of as a predictor of sarcopenia and mortality in idiopathic pulmonary fibrosis

**DOI:** 10.3389/fnut.2024.1440402

**Published:** 2024-12-04

**Authors:** Rocío Fernández-Jiménez, Eva Cabrera-Cesar, Alicia Sanmartín-Sánchez, Ana Sánchez-Garcia, Francisco Espildora-Hernandez, Isabel Vegas-Aguilar, Maria del Mar Amaya-Campos, Patricia Guirado-Pelaez, Victor Simón-Frapolli, Mora Murri, Lourdes Garrido-Sánchez, Lorena Piñel-Jimenez, Miguel Benítez Cano-Gamonoso, Javier López-García, Belén Gómez-Rodríguez, Jose Luis Velasco-Garrido, Francisco J. Tinahones, José Manuel García-Almeida

**Affiliations:** ^1^Department of Endocrinology and Nutrition, Virgen de la Victoria University Hospital, Malaga, Spain; ^2^Instituto de Investigación Biomédica de Málaga y Plataforma en Nanomedicina-IBIMA Plataforma BIONAND, Malaga, Spain; ^3^Department of Medicine and Dermatology, Málaga University, Malaga, Spain; ^4^Department of Endocrinology and Nutrition, Quironsalud Málaga Hospital, Malaga, Spain; ^5^Department of Neumology, Virgen de la Victoria University Hospital, Málaga, Spain; ^6^Department of Endocrinology and Nutrition, Son Espases Universitary Hospital, Carretera de Valldemossa, Palma, Spain; ^7^Department of Neumology, Carlos de Haya Regional University Hospital, Malaga, Spain; ^8^Department of Endocrinology and Nutrition, Hospital Universitario Virgen de la Victoria, CIBEROBN, Carlos III Health Institute (ISCIII), University of Málaga, Malaga, Spain; ^9^Heart Area, Victoria Virgen University Hospital; Instituto de Investigación Biomédica de Málaga y Plataforma en Nanomedicina-IBIMA Plataforma BIONAND, Malaga, Spain

**Keywords:** idiopathic pulmonary fibrosis, sarcopenia, morphofunctional assessment, quality of life, mortality

## Abstract

**Introduction:**

Idiopathic pulmonary fibrosis (IPF) is a progressive lung disease often complicated by sarcopenia, significantly impacting patient outcomes. This study investigates the prevalence and clinical implications of sarcopenia in IPF patients using morphofunctional assessment methods.

**Materials and methods:**

Eighty-four IPF patients (predominantly male) were evaluated for sarcopenia using the European Working Group on Sarcopenia in Older People 2 (EWGSOP2) criteria. Assessments included bioelectrical impedance vectorial analysis (Nutrilab, Akern), handgrip strength (HGS), Timed Up and Go test (TUG), and nutritional ultrasound (NU) measurements of rectus femoris and abdominal adipose tissue. Statistical analysis was performed (version 2.3.28 for macOS) to obtain sarcopenia cut-off points for the different techniques, and then the predictive capacity of these values for survival was analyzed using a Kaplan–Meier curve.

**Results:**

Sarcopenia was prevalent in 20.2% of the cohort. Sarcopenic patients exhibited significantly lower forced vital capacity (FVC) (2,142 mL vs. 2745.6 mL, *p* < 0.05), higher GAP stages (p < 0.05), and worse quality of life (SGRQ impact scores: 45.2 vs. 27.5, *p* < 0.05). The identified cutoff values were 2.94 cm^2^ for RFCSA, 9.19 s for TUG, and 1.08 cm for the RF-Y-axis and body cell mass (BCM) cutoff of 25.4 kg. Kaplan–Meier analysis indicated a higher hazard ratio (HR) for mortality in sarcopenic patients. Specifically, RFCSA sarcopenia patients had a 2.37 times higher risk of events (HR = 2.37, 95% CI: 1.02–5.48, *p* = 0.045), and TUG sarcopenia presented a 4.89 times higher risk of adverse events (HR = 4.89, 95% CI: 1.43–16.70, *p* = 0.011).

**Conclusion:**

Sarcopenia is prevalent in IPF patients and is associated with greater disease severity and reduced quality of life. RFCSA, BCM, and TUG are good predictors of sarcopenia and 12-month mortality, improving the prognostic value of classical diagnostics based on EWGSOP2 criteria. Despite limitations such as a predominantly male sample and cross-sectional design, the findings emphasize the importance of early detection and targeted interventions. Future research should focus on longitudinal studies to better understand sarcopenia progression in IPF and evaluate the efficacy of various therapeutic approaches.

## Background

Idiopathic pulmonary fibrosis (IPF) is the most common chronic and progressive interstitial lung disease (ILD) with unknown etiology. IPF patients often experience a progressive loss of muscle mass and strength, a condition known as sarcopenia, which has gained increasing attention in clinical research. The condition is considered idiopathic because its exact cause is not yet fully understood, although it is thought to be due to a combination of genetic and environmental factors (1). IPF mainly affects older adults and is characterised by symptoms such as shortness of breath, dry cough and fatigue. Over time, the disease can lead to a progressive decline in lung function and, in advanced stages, can become disabling and even fatal.

Nutrition and metabolism have been the subject of extensive scientific research in chronic obstructive pulmonary disease (COPD), but clinical awareness of the impact of dietary habits, nutritional status and nutritional interventions on the incidence, progression and outcome of COPD is limited. A multidisciplinary task force was set up by the European Respiratory Society to summarise the evidence and describe current practice in nutritional assessment and therapy in COPD (2, 3).

Respiratory distress and associated dyspnoea in IPF patients, as well as the use of new anti-fibrotic treatments that cause malabsorption, can make it difficult to eat adequately, leading to insufficient caloric intake and weight loss (4). Sarcopenia, characterized by the loss of muscle mass and decline in muscle function, can significantly impact the quality of life and outcomes of idiopathic pulmonary fibrosis patients (5, 6). Assessing and monitoring sarcopenia in this patient population is essential for enhancing disease management and designing targeted interventions (7).

Morpho-functional assessment, which provides parameters from bioelectrical impedance analysis (BIVA), nutritional ultrasound or functional tests such as lifting and walking or hand grip strength, have been identified as potential predictors of sarcopenia and mortality in different pathologies (8–15). These new techniques provide more valuable information than classical measures such as Body Mass Index (BMI) to identify potential predictors of sarcopenia in this population (16, 17). Furthermore, several studies suggest that these techniques are good predictors of mortality, and sarcopenia is associated with mortality. Therefore, identifying sarcopenia in these patients would give us a prognosis for survival in this group of patients (18–21).

Expanding on this knowledge, our study seeks to delve deeper into the potential utility of these measures as predictors of sarcopenia in IPF patients. By gaining a thorough understanding of the role of these parameters, we aim to contribute to the development of more effective management strategies and improved outcomes for individuals living with this debilitating condition. This study aims to investigate the utility of these measures in detecting and monitoring sarcopenia in IPF patients.

## Materials and methods

### Study design

A prospective, observational bicenter study of routine clinical practice was conducted at the nutrition unit of the Endocrinology and Nutrition Unit of the Virgen de la Victoria University Hospital. Patients diagnosed with idiopathic pulmonary fibrosis at various stages were included from our hospital and the Regional University Hospital of Málaga. All patients diagnosed with idiopathic pulmonary fibrosis who were being seen in the outpatient clinics of both hospitals were assessed, and those who met the inclusion criteria were selected. The assessments were conducted in an equal manner for all patients, at the same time of day, using the same techniques, and without any influence from the evaluator, as all measurements were carried out by the same specialist. Informed consent was obtained from all participants before they joined the study, which adhered to the Declaration of Helsinki and was approved by the Ethics Committee of Málaga on 5 April 2022 (reference number 1743-N-21). All enrolled patients met the inclusion criteria for idiopathic pulmonary fibrosis and provided informed consent, while none met any exclusion criteria such as refusal to participate or inability to undergo BIVA measurement due to specific reasons like ethnicity-related factors, extensive skin lesions, fluid extravasation, local hematomas, amputation or a life expectancy fewer than three months. The patient selection process is illustrated in a flow chart diagram (Supplementary Figure S1).

### Anthropometric and body composition measurements

Bioimpedance measurement, patients’ body weight and standing height were recorded. Weight was measured using a scale with 100 g sensitivity, and height was measured using a laser height rod with 2 mm sensitivity.

### Bioelectrical impedance vectorial analysis (BIVA^®^)

Body-composition analysis was performed using a 50 kHz phase-sensitive impedance analyser (V 101 Whole Body Bioimpedance Vector Analyzer, AKERN, Florence, Italy) that delivers 800 μA using tetrapolar electrodes positioned on the right hand and foot. All BIVA measurements were obtained with the patient in a supine position on a hospital bed. To stabilize BIVA values [±2 *Ω* for Resistance (R) and ± 1 Ω for Xc (Reactance)], the patient remained in a supine position for five minutes before obtaining BIVA measurements, as fluid shifts occur after moving from standing to recumbency and directly affect R and Z values. BIVA measurements of patients were standardized for sex and age using data from healthy Italian adults (22). PA is expressed in degrees as arctan (Xc/R) x (180o/*π*). An individual standardized PA value (SPA) was determined from the sex- and age-matched reference population value by subtracting the reference PA value from the observed patient PA value and dividing the result by the respective age- and sex-reference standard deviation (SD). The technical accuracy of the BIVA instrument was daily assessed using a precision circuit supplied by the BIVA device manufacturer (AKERN, Florence, Italy). All measured R and Xc values were consistently ±1 *Ω* of the 385 Ohm reference value. *In vivo* reproducibility of the BIVA measurements was also determined, with coefficients of variation (CV) of 1–2% for R and Xc (16, 23).

### Nutritional ultrasound (NU^®^)

Ultrasound measurements of the unilateral (right) rectus femoris were performed at the reference centre by an experienced medical sonographer, blinded to clinical data and other results of the nutritional assessment, using a commercially available portable ultrasound system with a 4–10 cm linear tube (Mindray Z60, Madrid, Spain). Abdominal and anterior thigh muscle measurements were performed with the patient in the supine position with knees extended and relaxed. A 7.5–10 kHz linear ultrasound probe was used. The acquisition site was located two-thirds of the way along the femur, measured between the anterior superior iliac spine and the upper edge of the patella. The transducer was placed perpendicular to the long axis of the femur, with excessive use of contact gel and minimal pressure to avoid muscle compression. All parameters were taken as the average of three consecutive measurements in the dominant leg. We measured the transversal axis of the cross-sectional area (CSA) in cm2; the X and Y axes in cm, which corresponded to the linear measurement of the distance between the muscle limits of the rectus femoris (lateral and anteroposterior); the X/Y axis ratio; and the total adipose tissue in cm.

A trained physician performed the ultrasound with the probe perpendicular to the longitudinal and transverse axes of the RF. The main parameters measured were rectus femoris cross-sectional area (RF-CSA), rectus femoris circumference (RF-CIR) and leg subcutaneous adipose tissue (L-SAT). Each parameter was measured three times and the mean was calculated.

For the assessment of abdominal adipose tissue, measurements were taken at the midpoint between the xiphoid appendage and the umbilicus. This included total subcutaneous abdominal fat (T-SAT), superficial subcutaneous abdominal fat (S-SAT) and visceral fat (VAT), all measured in centimeters.

### Functional assessment

Handgrip strength (HGS) was measured using a JAMAR hand dynamometer (Asimow Engineering Co., Los Angeles, CA, United States). Grip strength was determined in a seated position with the elbow flexed at 90 degrees in the dominant hand. Patients were instructed to perform three maximal isometric contractions with brief pauses between measurements, and the median value was recorded. Test Up and Go was selected to evaluate functional capacity. Walk 3 was performed with the patient seated in a chair. The time taken to get up, walk 3 meters, turn around, walk another 3 meters, and sit back down was measured in seconds. All patients underwent spirometry, lung volume, and diffusion capacity testing at the Pulmonary Function testing lab at the time of enrollment, and at 6 and 12-month follow-up. St. George’s Respiratory Questionnaire (SGRQ) was administered that assesses three components—Symptoms, Activity, and impact on daily life. Enrollment Measures of FVC, DLCO, SGRQ are considered the baseline value.

### Clinical outcomes: assessment of sarcopenia and mortality

Probable sarcopenia and sarcopenia were diagnosed according to EWGSOP2 (European Working Group on Sarcopenia in Older People 2); i.e. a HGS <16 kg for women and < 27 kg for men, indicated probable sarcopenia. When combined with low muscle mass; i.e. ASMM<20 kg for men and ASM < 15 kg for women or < ASMI <5.5 kg/m2 for women and < 7.0 kg/m2 for men (measured by BIVA) the diagnosis of sarcopenia was confirmed here denoted sarcopenia-BIVA (24).

Mortality was defined as death from all causes up to 35 months after the first assessment of the patient. However, for the purpose of the survival curves, the first year of follow-up was considered to evaluate the effect of mortality across the different techniques used in the study.

### Statistical analyses

Data analysis was primarily conducted using the JAMOVI software (version 2.3.28 for macOS). The findings are displayed as the mean ± standard deviation for continuous variables and in numbers (percentages) for categorical variables. A Student t-test or Wilcoxon test was executed based on the normality of the variables examined in this research. Pearson correlation coefficients were employed to assess relationships between variables. Statistical significance was established at a threshold of *p* < 0.05, with significance determined using either a p < 0.05 test or the Chi-squared test.

The evaluation of the predictive property of muscle mass variables was based on the receiver operating characteristic curve (ROC) and AUC (area under the curve). The diagnostic accuracy of RF-CSA, RF-Y-Axis, BCM, timed up and go test (TUG) and Forced Vital Capacity (FCV) in predicting sarcopenia was assessed using receiver operating characteristic curves and the area under the curve. Sensitivity versus specificity plots were used to calculate AUC values for each measurement. Optimal cut-off values were determined by identifying the point where sensitivity and specificity converge.

Lineal and logistic regression analysis was utilized in this research. The binomial logistic regression analysis yielded the odds ratio along with 95% confidence intervals. To determine 12-month survival probabilities, we used the Kaplan–Meier estimator. The cut-off point from the ROC curve for predicting sarcopenia was applied to assess the risk of mortality related to sarcopenia. This approach also helped evaluate differences in sarcopenia rates based on BIVA, NU, and functional test cut-off values. Kaplan–Meier survival curves were compared using the log-rank test to identify significant differences, with *p* < 0.05 considered statistically significant.

## Results

### Characteristics of the patient

In our study, we examined a cohort of 84 individuals, with a gender imbalance noted in the composition of the participants: a predominant 83.5% (71 individuals) and a mean age of 71.0 years (7.22 years). Table 1 presents a comparative analysis of Demographic and anthropometric variables between sarcopenic and non-sarcopenic patients with idiopathic pulmonary fibrosis diagnosis.

**Table 1:** Comparison in demographic and anthropometric characteristics between sarcopenic and non-sarcopenic at IPF diagnosis.

	All	Non-Sarcopenic	Sarcopenic	*p* value
	*N* = 84	*N* = 67	*N* = 17	
Demographic and anthropometric variables				
Age (years)	71.0 (7.22)	70.2 (7.01)	74.3 (7.50)	<0.05**
Gender (male)	85 (83.5%)	65 (77.4%)	5 (6%)	<0.001***
BMI (kg/m^2^)	27.6 (3.47)	27.5 (3.46)	28.2 (3.47)	<0.466
Weight (kg)	79.1 (12.5)	80.5 (13.1)	73.6 (8.64)	<0.05*
BIVA				
PA (°)	4.83 (0.764)	4.89 (0.79)	4.58 (0.63)	0.133
SPA	−0.96 (0.950)	−1.03 (0.97)	−0.66 (0.81)	0.161
Nutrition	761 (162)	790 (164)	648 (96.5)	<0.001***
Hidration	74.6 (2.39)	74.7 (2.51)	74.3 (1.89)	0.486
BCM (kg)	25.9 (5.12)	27.0 (4.96)	21.5 (3.19)	<0.001***
Nak	1.17 (0.187)	1.18 (0.191)	1.14 (0.177)	0.440
FFMI (kg/m^2^)	19.2 (1.70)	19.5 (1.69)	18.1 (1.07)	<0.001***
FMI (kg/m^2^)	8.43 (2.82)	8.02 (2.45)	10.2 (3.59)	<0.001***
BCMI (kg/m^2^)	9.10 (1.89)	10.2 (1.58)	7.95 (1.43)	<0.001***
SMI (kg/m^2^)	8.90 (1.18)	9.26 (0.969)	7.58 (0.974)	<0.001***
ASMI(kg/m^2^)	20.4 (3.31)	21.3 (3.11)	17.1 (1.67)	<0.001***
Echography exploration				
RF-CSA (cm^2^)	3.36 (0.995)	3.59 (0.97)	2.48 (0.46)	<0.001***
RF-CIR (cm)	8.21 (1.10)	8.51 (0.95)	7.00 (0.82)	<0.001***
RF-X-axis (cm)	3.45 (0.485)	3.57 (0.43)	3.01 (0.44)	<0.001***
RF-Y axis (cm)	1.12 (0.279)	1.16 (0.28)	0.95 (0.18)	<0.001***
L-SAT (cm)	0.78 (0.526)	0.66 (0.40)	1.25 (0.68)	<0.001***
T-SAT (cm)	1.69 (0.715)	1.53 (0.63)	2.28 (0.70)	<0.001***
S-SAT (cm)	0.73 (0.298)	0.65 (0.24)	1.01 (0.29)	<0.001***
VAT (cm)	0.65 (0.306)	0.62 (0.26)	0.74 (0.43)	0.179
Functional measurement				
HGS mean (kg)	33.0 (10.0)	36.3 (8.27)	20.2 (4.61)	<0.001***
TUG (s)	7.64 (1.86)	7.32 (1.54)	8.88 (2.52)	<0.001***

Data are expressed as mean ± standard deviations or percentage. Groups were divided by non-sarcopenic vs sarcopenic variable. Asterisk indicates significant difference between groups, according to the t-student test (Chi squared test was used for variables expressed as percentage) (***p < 0.001, **p < 0.01, *p < 0.05). BCM: Body cell mass; BCMI: BCM index; BMI: Body mass index; BIVA: Bioelectrical vectorial Impedance Analysis; CRP: C reactive protein; FM: Fat mass; FMI: FM index; FFMI: Fat-free mass index; HGS: Hand grip strength; PA: Phase angle; RF- CIR: circumference of quadriceps rectus femoris; RF-CSA: rectus femoris cross-sectional area; SAT: subcutaneous adipose fat of leg (L), superficial (S) and total (T) abdominal; SMI: Skeletal muscle index; SPA: Standardized phase angle; TSH: Thyroid-stimulating hormone; TUG: Timed Up & Go.

The data show that sarcopenic patients were older on average compared to non-sarcopenic patients (74.3 years vs. 70.2 years, *p* < 0.05). A higher proportion of the total and non-sarcopenic groups were male, and a higher proportion of sarcopenic were female. BMI did not differ significantly between groups, suggesting that BMI alone may not be a reliable indicator of sarcopenia in patients with IPF (*p* value <0.466). The non-sarcopenic patients weighed more on average compared to sarcopenic patients (80.5 kg vs. 73.6 kg).

The study also analyzed bioelectrical impedance variables, nutritional variables, and body composition measurements, resulting in the following significant findings: The non-sarcopenic IPF patients had higher nutritional measurements compared to sarcopenic patients (790 vs. 648, *p* < 0.05), Non-sarcopenic patients exhibited a higher BCM (body mass cell) and BCMI (body mass cell index) compared to sarcopenic patients. Both indices were significantly different between the groups, with sarcopenic patients having lower FFMI (fat free mass index), SMI (squeletal mass index) and ASMI (appendicular squeletal mass index) and higher FMI (fat mass index), *p* < 0.05.

### Comparison in clinicopathological variables between sarcopenic and non-sarcopenic

The Table 2 provides a comparison of clinical pathological variables between non-sarcopenic and sarcopenic patients in the context of IPF. There was no statistical difference in the distribution of treatments between the groups, with Ofev and Pirfenidone being the primary treatments. The mean FCV (ml) value was 2,620 in total, significantly higher in non-sarcopenic (2745.6) compared to sarcopenic (2142), with a *p*-value of <0.05**, indicating significant differences. The St. George’s Respiratory Questionnaire was used to measure symptoms and impairment. Sarcopenic patients reported higher impairment compared to non-sarcopenic patients (*p* < 0.05). When assessed with the Subjective Global Assessment (SGA) and the Global Leadership Initiative on Malnutrition criteria (GLIM), no significant differences were observed with SGA, while with GLIM, sarcopenic patients had a higher risk of malnutrition compared to non-sarcopenic patients (p < 0.05). There was a non-significant trend towards higher mortality rates among non-sarcopenic patients (*p* = 0.106).

**Table 2:** Comparison in clinicopathological and malnutrition variables between sarcopenic and non-sarcopenic at IPF diagnosis.

	All	Non-sarcopenic	Sarcopenic	*p* value
	*N = 84*	*N = 67*	*N = 17*	
Clinicopathological variables				
Respiratory treatment				0.830
Without treatment	1 (1.4%)	1 (1.4%)	0 (0.0%)	
Ofev	49 (68.1%)	38 (53.5%)	11 (15.5%)	
Pirfenidona	22 (30.6%)	17 (23.9%)	4 (5.6%)	
GAP stage				<0.05**
Stage I	27 (32,1%)	18 (21,4%)	9 (10.7%)	
Stage II	41 (47,6%)	34 (40.5%)	6 (7.1%)	
Stage III	17 (20,2%)	15 (17.9%)	2 (2,4%)	
GAP Score	4 (1,8)	4 (1,8)	3 (1,6)	0.181
DLCO (L)	49.6 (17.3)	47.8 (14.9)	56.6 (23.1)	0.096
KCO (L)	79 (21.2)	78.4 (20.6)	82.4 (23.4)	0.542
FCV (ml)	2,620 (16.4)	2745.6 (723)	2,142 (800.2)	<0.05**
SGRQ Sintoms	34.8 (19.5)	33.6 (20.8)	38.9 (14.4)	0.375
SGRQ Activity	34 (7.38%)	17 (7.36%)	17 (7.39%)	0.150
SGRQ imp	31.4 (27.7)	27.5 (26.0)	45.2 (30.0)	<0.05**
Malnutrition (SGA)				0.381
Non malnutrition	15 (18.5%)	11 (13.8%)	4 (5%)	
Moderate risk	50 (61.7%)	41 (51.2%)	8 (10%)	
Hight risk	16 (19.8%)	11 (13.8%)	5 (6.3%)	
Malnutrition (GLIM)				<0.05**
Non malnutrition	18 (21.4%)	18 (21.4%)	0 (0.0%)	
Malnutrition	66 (78.6%)	49 (58.1%)	17 (20,5%)	
Mortality				0.106
No	62 (72,9%)	46 (54.8%)	15 (17.9%)	
Yes	23 (27.1%)	21 (25%)	2 (2.4%)	

Data are expressed as mean ± standard deviations or percentage. Groups were divided by non-sarcopenic vs sarcopenic variable. Asterisk indicates significant difference between groups, according to Chi squared test for dichotomic variables (***p < 0.001, **p < 0.01, *p < 0.05). GAP: gender (G), age (A) and physiology (P) index; FCV: Forced Vital Capacity; DLCO: pulmonary diffusion of carbon monoxide; KCO: Carbon monoxide transfer coefficient; SGRQ: Saint George’s Respiratory Questionnaire; SGA: Subjective Global Assessment; GLIM: Global Leadership Initiative on Malnutrition.

### Correlation analysis between muscle measures: BIVA, NU and functional test (HGS and TUG) with sarcopenia

**Figure 1:**
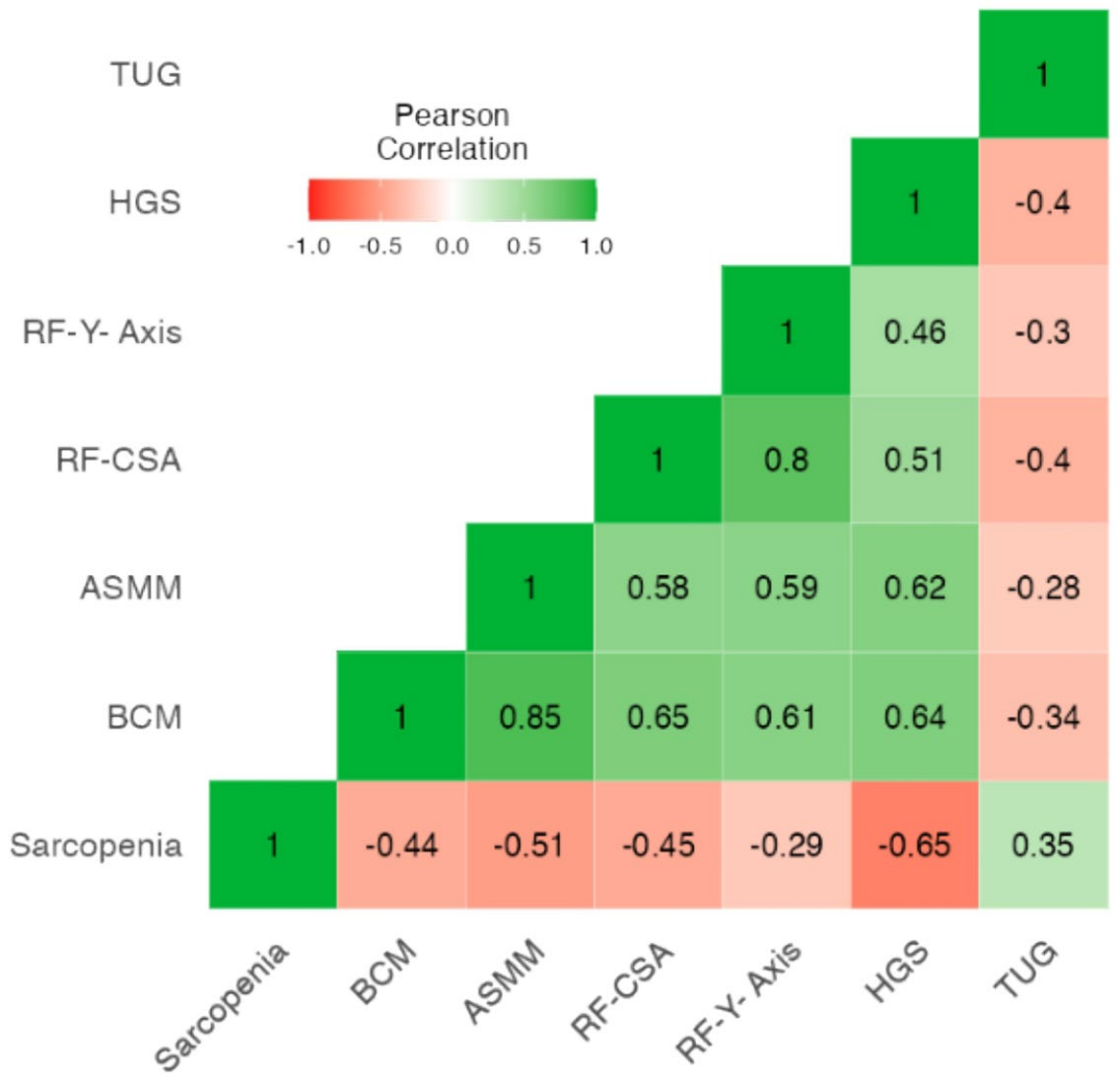
Heatmap correlation plots are presented to show association between body composition (BIVA, NU, and functional tests) with sarcopenia of all participants. ASMM: appendicular squeletal mass muscle; Pha: Phase angle; BCM: Body cell mass; RF-CSA: rectus femoris cross sectional area; RF-Y-Axis: rectus femoris Y axis, HGS: Hang grip strength; TUG: up and go test.

The correlation matrix (Figure 1) shows significant negative correlations between sarcopenia and handgrip strength (HGS) (*r* = −0.653, *p* < 0.001), body cell mass (BCM) (*r* = −0.433, *p* < 0.001), appendicular skeletal muscle mass (ASMM) (*r* = −0.509, *p* < 0.001), and rectus femoris cross-sectional area (RF-CSA) (*r* = −0.451, *p* < 0.001). This indicates that as sarcopenia severity increases, these muscle mass and strength parameters decrease.

HGS shows strong positive correlations with BCM (*r* = 0.638, *p* < 0.001), ASMM (*r* = 0.619, *p* < 0.001), and RF-CSA (*r* = 0.508, *p* < 0.001), suggesting that greater handgrip strength is associated with higher muscle mass.

The rectus femoris Y-axis (RF-Y-Axis) is positively correlated with BCM (*r* = 0.606, *p* < 0.001), ASMM (*r* = 0.585, *p* < 0.001). The rectus femoris cross-sectional area (RF-CSA) shows strong positive correlations with handgrip strength (*r* = 0.508, *p* < 0.001), body cell mass (BCM) (*r* = 0.643, *p* < 0.001), and appendicular skeletal muscle mass (ASMM) (*r* = 0.581, *p* < 0.001). These correlations indicate that RF-CSA is a reliable indicator of muscle mass and strength, making it valuable for assessing sarcopenia in patients.

Additionally, TUG is negatively correlated with HGS (*r* = −0.402, *p* < 0.001), BCM (*r* = −0.344, *p* = 0.001), and RF-CSA (*r* = −0.398, *p* < 0.001), showing that poorer TUG performance is associated with lower muscle mass and strength.

### Cut-off points of morphofunctional parameters: bioelectrical, ultrasound and functional in sarcopenia prediction in patients with IPF

We have further explored the predictive power of various nutritional assessment methods regarding sarcopenia in patients with IPF (Table 3). The BMI presented a cut-off value of 29.1 kg/m^2^, but exhibited a moderate AUC of 0.559, suggesting limited predictive value (sensitivity and specificity at 47.06 and 68.66% respectively, *p* < 0.05).

**Table 3:** Predictive value of nutritional assessment methods on sarcopenia in patients with idiopathic pulmonary patients.

Variables	Cut-off▴	AUC	Sensitivity	Specificity	*p* value
BIVA and classical parameters					
BCM	25.4	0.822	61.19%	94.12%	<0.05**
Nutritional Ultrasound					
RF-CSA	2.94	0.833	75.76%	88.24%	<0.05**
RF-Y-Axis	1.08	0.730	60.61%	82.35%	<0.05**
Functional test					
TUG	9.19	0.714	47.06%	87.69%	<0.05**
Pulmonary function					
FCV (ml)	2,500	0.722	61.02%	65.47%	<0.05**

Receiver operating characteristic (ROC) and Cut-off without adjusting for variables for the nutritional assessment methods and sarcopenia in patients with idiopathic pulmonary patients. BCM: Body cell mass; RF-CSA: rectus femoris cross sectional area; RF-Y-Axis: rectus femoris Y axis, TUG: up and go test; FCV: Forced Vital Capacity.

**Figure 2:**
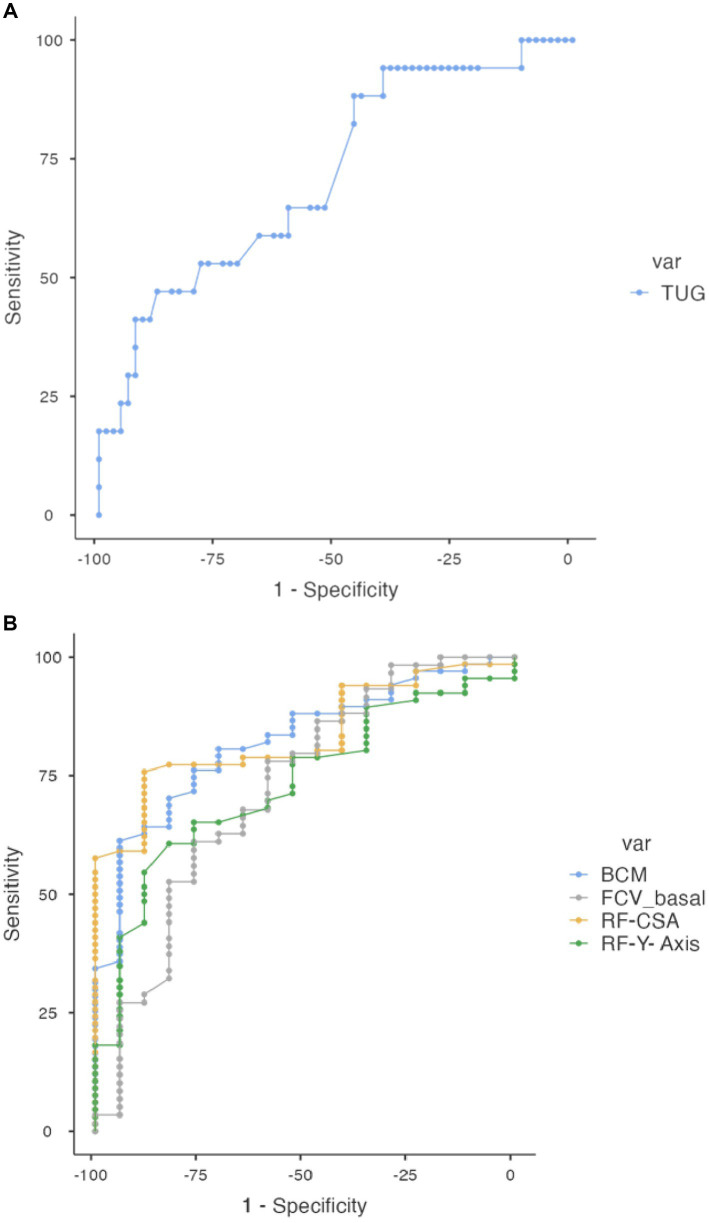
Comparative analysis of ROC curve for variables associated with sarcopenia. **(A)** ROC curve for TUG with established cut-off point. **(B)** ROC curves for other variables including BCM, FCW_basal, RF-CSA, and RF-Y Axis, with established cut-off points.

BCM, on the other hand, had a cut-off of 25.4 kg, with a significantly higher AUC of 0.822, indicating a strong predictive ability (Sensitivity and specificity 61.19 and 94.12% respectively, *p* < 0.05), shows in Figure 2.

In the field of NU, the RF-CSA showed a cut-off of 2.94 cm^2^ and an AUC of 0.833, highlighting its robust predictive capability (sensitivity and specificity were marked at 75.76 and 88.24%, with a highly significant *p*-value). The RF-Y-Axis variable presented a cut-off at 1.08 cm and an AUC of 0.730 (sensitivity and specificity of 60.61 and 82.35%, *p* < 0.05).

Functional tests represented by the TUG exhibited a cut-off of 9.19 s and an AUC of 0.714 (sensitivity of 47.06% specificity of 87.69%, *p* < 0.05). For pulmonary function, the Forced Vital Capacity (FCV) with a cut-off of 2,500 mL shows an AUC of 0.722, sensitivity of 61.02%, and specificity of 65.47%, indicating moderate predictive ability, with a *p*-value of <0.05.

The binomial logistic regression analysis identified significant predictors of sarcopenia in patients with idiopathic pulmonary disease. The model demonstrated good fit with a deviance of 54.0 and an AIC of 64.0, explaining approximately 35.9% of the variance (McFadden’s R^2^). RF-CSA (Estimate = −1.3593, SE = 0.5989, *p* = 0.023, OR = 0.257, 95% CI [0.0794, 0.831]) and BCM (Estimate = −0.2230, SE = 0.1030, *p* = 0.030, OR = 0.800, 95% CI [0.6539, 0.979]) were significant predictors, indicating that higher values of these variables reduce the odds of sarcopenia. Age was not a significant predictor (Estimate = 0.0175, SE = 0.0557, *p* = 0.754, OR = 1.018, 95% CI [0.9123, 1.135]). BMI (obesity) was marginally significant (Estimate = 1.5040, SE = 0.8586, *p* = 0.080, OR = 4.500, 95% CI [0.8363, 24.212]), suggesting a potential increase in sarcopenia odds with higher BMI. Collinearity diagnostics indicated no significant multicollinearity among the predictors, with VIF values close to 1. The model’s overall accuracy was 81.9%, with a specificity of 92.4% and a sensitivity of 41.2%. The AUC of 0.890 indicates good discriminatory ability (Table 4).

**Table 4:** Model coefficients on sarcopenia in patients with idiopathic pulmonary patients.

95% confidence interval							
Predictor estimate		SE	Z	*P* value	Odds ratio lower upper		
Intercept	6.29	5.17	1.21	0.223	543.9	0.021	138e+7
RF-CSA	−1.35	0.59	−2.27	0.023	0.25	0.079	0.831
BCM	−0.22	0.10	−2.16	0.030	0.80	0.65	0.97
Age	0.017	0.055	0.31	0.75	1.01	0.91	1.135
BMI-Obesity							
0–1	1.50	0.85	1.75	0.080	4.50	0.83	24.21

BCM: Body cell mass; RF-CSA: rectus femoris cross sectional area; BMI: Body mass index. Estimates represent the log odds of “Sarcopenia = 1” vs. “Non-Sarcopenia = 0”.

### Kaplan–Meier survival curve of 12 month-mortality in IPF patients with sarcopenia and morphofunctional assessment techniques

For idiopathic pulmonary fibrosis patients in the study, sarcopenia as measured by body composition markers and body mass index exhibited varying impacts on survival.

**Figure 3:**
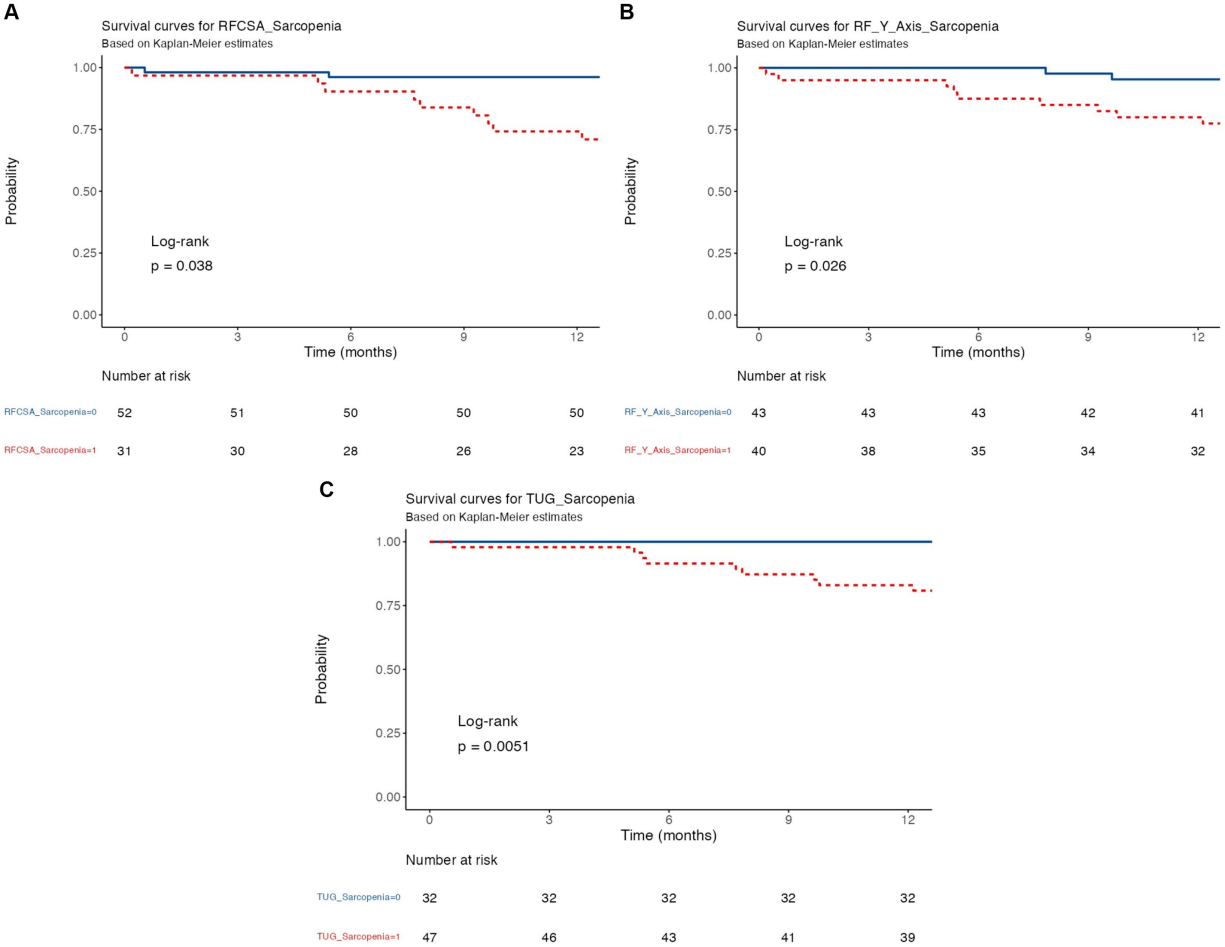
Kaplan–Meier curves for the variables included in the study. (A) Kaplan- Meier curve RF-CSA Sarcopenia. (B) Kaplan–Meier curve RF-Y-Axis sarcopenia; (C) Kaplan–Meier curve TUG sarcopenia. RF-CSA: rectus femoris cross sectional area; RF-Y-Axis: rectus femoris Y axis, TUG: up and go test.

The survival analysis indicated that patients with RF-CSA Non-Sarcopenia had a 74% 12-month survival rate compared to 96% for those with RF-CSA Sarcopenia, with median survival times of at least 35.3 months and 18.1 months, respectively (Figure 3). The Cox regression analysis showed that RF-CSA Sarcopenia patients had a 2.37 times higher risk of an event (HR = 2.37, 95% CI: 1.02–5.48, *p* = 0.045) (Table 5).

**Table 5:** Univariable analysis of survival and hazard ratios for sarcopenia across different variables at 12 months in patients with idiopathic pulmonary patients.

Variable	Levels	All	HR (Univariable)	95% CI (HR)	*p*-value	Time (months)	Survival (%)	95% CI (survival)
RFCSA SARCOPENIA	Non-sarcopenia	52				12	96.2	91.1–100.0
	Sarcopenia	31	2.37	1.02–5.48	0.045	12	74.2	60.3–91.3
RF_Y_Axis sarcopenia	Non-sarcopenia	43				12	95.3	89.3–100.0
	Sarcopenia	40	2.67	1.09–6.55	0.032	12	80.0	68.5–93.4
TUG sarcopenia	Non-sarcopenia	32				12	100.0	100.0–100.0
	Sarcopenia	47	4.89	1.43–16.70	0.011	12	83.0	72.9–94.4

RF-CSA: rectus femoris cross-sectional area; RF_Y_Axis: rectus femoris Y Axis; TUG: Timed Up & Go; HR hazard ratio; CI confidence interval.

When RF-Y-Axis Sarcopenia was present, there was a 2.67 times higher risk of adverse events (HR = 2.67, 95% CI: 1.09–6.55, *p* = 0.032). The 12-month survival rate was 95% for patients without sarcopenia, compared to 80% for those with sarcopenia.

Lastly, we observe the analysis for TUG sarcopenia. When TUG Sarcopenia is present, there is a 4.89 times higher risk of adverse events (HR = 4.89, 95% CI: 1.43–16.70, *p* = 0.011). The 12-month survival rate is 100% for patients without sarcopenia, compared to 83% for those with sarcopenia.

## Discussion

The results presented in this study provide a comprehensive insight into the prevalence and impact of sarcopenia in patients with idiopathic pulmonary fibrosis (IPF). Using the European Working Group on Sarcopenia in Older People 2 (EWGSOP2) criteria to determine sarcopenia, it has been demonstrated through a detailed analysis of clinicopathological variables that sarcopenia is not only common in this population but is also associated with greater disease severity and worse clinical outcomes. Specifically, the functional and nutritional evaluation of these patients revealed significant differences between those with and without sarcopenia, including the determination of cutoff values for rectus femoris cross-sectional area (RF-CSA) and Timed Up and Go (TUG) test. These findings highlight the importance of early detection and management of this condition to improve quality of life and potentially long-term outcomes in patients with IPF.

In this study, sarcopenia was identified in 20.2% of the cohort. This prevalence is consistent with findings from previous research (5) reported a sarcopenia prevalence of 22.9% in a similar IPF cohort, indicating that our results are in line with existing literature. However, Ohkubo et al. (25, 26) found a higher prevalence of 31.9% in their study, suggesting variability depending on the population and diagnostic criteria used.

The distribution of respiratory treatments (Ofev and Pirfenidone) did not differ significantly between sarcopenic and non-sarcopenic patients in our study (*p* = 0.830). This is consistent with previous findings, indicating that the choice of antifibrotic therapy may not directly influence the prevalence of sarcopenia (26) also found no significant differences in treatment distribution, suggesting that sarcopenia in IPF is independent of specific pharmacological interventions. However, the treatment of sarcopenia is crucial for patients with pulmonary fibrosis, as nintedanib has been shown in various studies to decrease appetite (27, 28) Additionally, it is now approved for progressive pulmonary fibrosis, making it a common treatment for most of these patients. Therefore, it is essential to pay special attention to the side effects and their impact on sarcopenia in patients with idiopathic pulmonary fibrosis.

Our study not found that sarcopenic patients had higher GAP stages. This differs with the findings of Faverio et al. (5) who reported that higher disease severity, as measured by the GAP index, was associated with sarcopenia. Additionally, we observed that forced vital capacity (FVC) was significantly lower in sarcopenic patients (2,142 mL) compared to non-sarcopenic patients (2745.6 mL), with a *p*-value of <0.05. This significant reduction in lung function in sarcopenic patients is consistent with previous studies (7, 29), emphasizing the impact of muscle wasting on respiratory capacity.

The nutritional assessment using GLIM criteria revealed significant differences, with a higher proportion of sarcopenic patients being at nutritional risk (*p* < 0.05). This is consistent with the findings of other articules, who reported that malnutrition (30, 31) and sarcopenia (6, 24) often coexist in chronic diseases, exacerbating overall morbidity. Our study also aligns with Faverio et al. (5) who found that malnutrition, assessed through various criteria, was more prevalent in sarcopenic IPF patients.

We identified the cut-off values for all parameters of BIVA, NU and functional test, comparing our findings with the DRECO study by de Luis Roman et al. (15) which assessed ultrasound cut-off values for rectus femoris in patients at nutritional risk, we find some parallels and distinctions. The DRECO study reported cut-off values ranging from 2.40 cm^2^ to 3.66 cm^2^ for the RF-CSA, which is in line with our RF-CSA cutoff of 2.94 cm^2^. However, the DRECO study’s context of hospitalized patients at nutritional risk differs from our cohort of IPF patients, highlighting the broader applicability of our findings specifically to IPF management.

Point-of-care ultrasonography (POCUS) has emerged as an invaluable tool enables clinicians to perform rapid, bedside evaluations of muscle mass and other parameters without the need for specialized radiology departments, thereby streamlining the diagnostic process and reducing healthcare costs. Studies have demonstrated that POCUS is highly effective in diagnosing a variety of conditions, with the added benefit of integrating sonographic findings with clinical examination at the patient’s bedside (32). This integration enhances the diagnostic accuracy and facilitates timely interventions, making POCUS an essential component of the morphofunctional assessment in sarcopenia.

In a recent study by Fujita et al. (33) on patients with IPF and sarcopenia, it was similarly found that sarcopenia is significantly associated with poorer physical performance and reduced quality of life. Their study identified that the distance covered in the six-minute walk test (6MWD) is an independent factor associated with sarcopenia, whereas our results demonstrated a significant correlation between sarcopenia and other functional tests, such as HGS and the TUG. Both our study and Fujita et al.’s highlight that patients with sarcopenia have lower scores on the St. George’s Respiratory Questionnaire (SGRQ), underscoring the negative impact of sarcopenia on the overall well-being of IPF patients.

Moreover, studies focusing on patients with COPD (34) have shown that the TUG test demonstrates good sensitivity and specificity in identifying patients with reduced exercise capacity, as measured by the 6MWT, with TUG times over 11 s indicating poor functional capacity. Improvements in TUG time were also significantly correlated with enhancements in 6MWD following pulmonary rehabilitation, further supporting the use of TUG as a valuable functional assessment tool in IPF patients. These findings collectively underscore the importance of comprehensive assessments that include not only muscle mass evaluation but also physical performance and emotional well-being in IPF patients, reinforcing the relevance of incorporating TUG into the clinical assessment of sarcopenia and its utility in predicting clinical outcomes in patients with chronic diseases (35, 36). The simplicity and effectiveness of the TUG make it an ideal tool for routine clinical practice, especially in settings with limited time and space, distinguishing it from the 6MWD.

In light of the growing recognition of sarcopenia as a critical factor in chronic diseases, the search for clinically applicable markers has intensified. A large-scale study conducted in the USA involving 11,761 participants identified elevated red cell distribution width (RDW) as a potential marker associated with sarcopenia. This finding has spurred further research in chronic lung diseases, where increased RDW has been linked to poor prognosis in idiopathic pulmonary fibrosis (IPF) and other chronic lung conditions (37–39). These results underscore the importance of identifying reliable and easily measurable biomarkers like RDW to enhance the clinical management of sarcopenia in patients with IPF, further supporting the integration of such markers into routine clinical practice for better prognostic assessment and individualized treatment strategies.

In summary, the integration of RFCSA and TUG test into routine assessments for IPF patients provides a comprehensive view of both muscle mass and functional mobility. These measures are crucial for early identification of sarcopenia and the implementation of targeted interventions. For example, resistance training programs aimed at increasing muscle mass and strength could be tailored based on RFCSA measurements. Similarly, rehabilitation programs focusing on improving mobility and reducing fall risk could be designed using TUG test results.

Although the difference in mortality between sarcopenic and non-sarcopenic patients was not statistically significant in our study (*p* = 0.106), likely due to the small number of sarcopenic patients, previous studies, such as those by Faverio et al. (5) and Ohkubo et al. (7, 40), did report higher mortality rates among sarcopenic patients, suggesting that sarcopenia may be a prognostic marker of poor outcomes in IPF.

This study has some limitations, despite its relatively small sample size and cross-sectional design, provides valuable insights into the prevalence and impact of sarcopenia in patients with idiopathic pulmonary fibrosis (IPF). Conducted at a bicenter with a predominantly male population, which is typical for IPF but may limit the generalizability of the results, the research might have limited applicability to a more diverse population. Additionally, while established methods like bioelectrical impedance analysis (BIVA) and handgrip strength (HGS) were employed, potential confounding factors and measurement inconsistencies were not fully accounted for. However, the study’s comprehensive approach, including the use of multiple diagnostic criteria (EWGSOP2), functional tests (HGS, TUG), and imaging techniques (RF-CSA), enhances the reliability of the findings.

The results align with existing literature, emphasizing the clinical relevance of early detection and management of sarcopenia in IPF. The identification of cut-off values for RF-CSA and TUG provides practical tools for clinicians, and the multifaceted assessment offers a holistic view of patient health, highlighting the need for interventions addressing both muscle mass and function. Future research should build on these strengths while addressing the study’s limitations to further advance the understanding of sarcopenia in chronic respiratory diseases.

## Conclusion

In conclusion, our study suggests that sarcopenia, specifically as measured through morphological muscle techniques such as the RF-CSA (NU) and functional tests as the TUG, are valuable predictors of survival in patients with idiopathic pulmonary fibrosis. These findings highlight the importance of considering muscle mass and function in the management and prognosis of IPF patients. Moreover, the presence of sarcopenia may indicate a broader deterioration in these patients, impacting their quality of life, respiratory function and mortality. The morphofunctional assessments are feasible techniques and can provide valuable information in clinical evaluation and therapeutic decision-making for IPF patients.

## Data Availability

The original contributions presented in the study are included in the article/Supplementary material, further inquiries can be directed to the corresponding authors.
