# Supplementary figures and images for: Rectus femoris cross sectional area and timed up and go test potential useful of as a predictor of sarcopenia and mortality in idiopathic pulmonary fibrosis
Source: Front Nutr. 2024 Dec 4;11:1440402. doi: 10.3389/fnut.2024.1440402 (PMC11652176; doi:10.3389/fnut.2024.1440402)

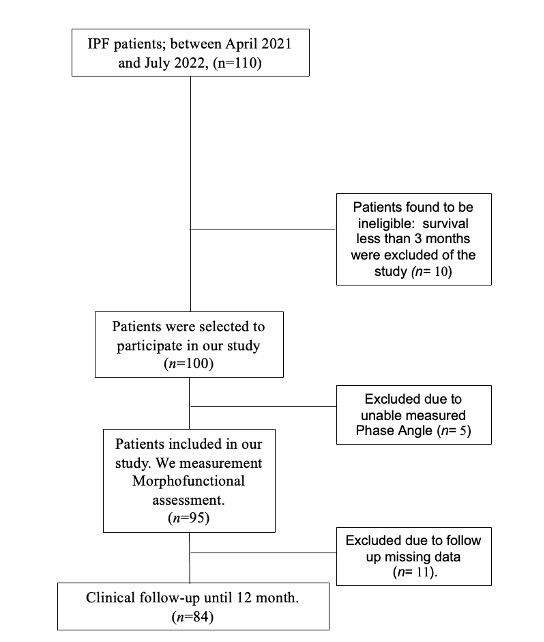

Supplement: Supplementary file 1 [file Image_1.JPEG]

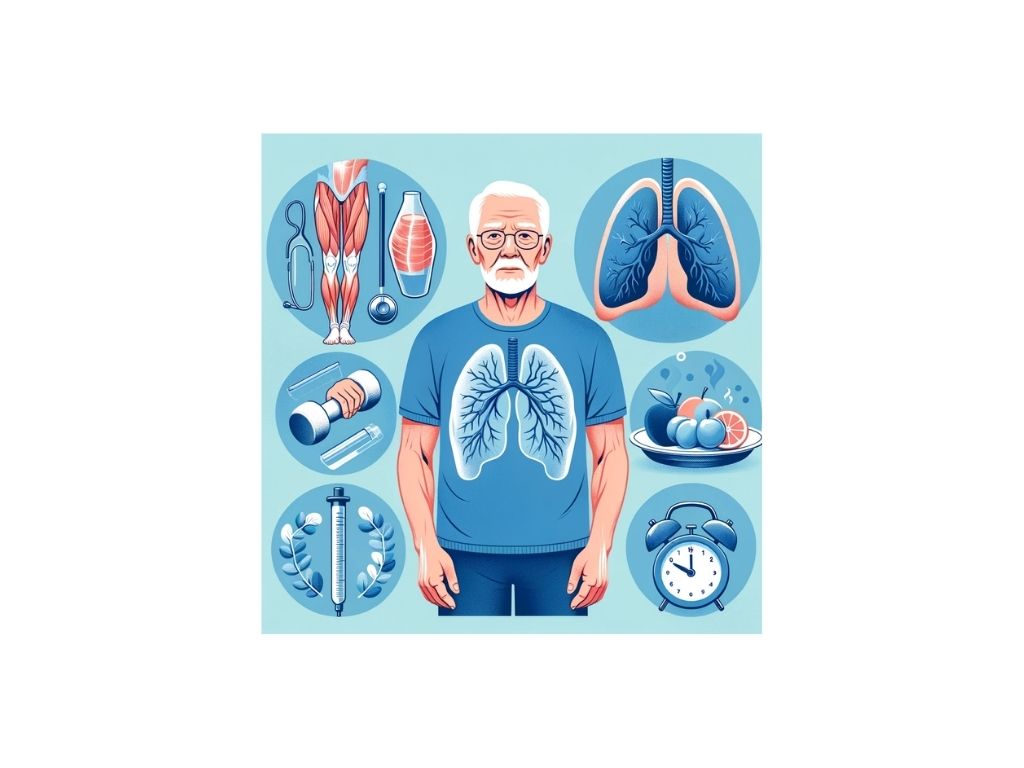

Supplement: Supplementary file 2 [file Image_2.JPEG]
